# Genetic Analysis Reveals Relationships Among Populations of *Puccinia triticina* from Henan Province of China

**DOI:** 10.3390/jof12070468

**Published:** 2026-06-25

**Authors:** Shuhe Wang, Yi Yang, Yuxin Gu, Jinhang Luo, Shengming Liu

**Affiliations:** 1 College of Horticulture and Plant Protection, Henan University of Science and Technology, Luoyang 471023, China; 18790199182@163.com (Y.Y.); 17634366637@163.com (Y.G.); ljh20021228@163.com (J.L.); liushengming@haust.edu.cn (S.L.); 2 Henan Province Engineering Technology Research Center of Green Plant Protection, Luoyang 471023, China

**Keywords:** *Puccinia triticina*, population structure, multilocus genotyping, wheat, leaf rust

## Abstract

Wheat leaf rust, caused by *Puccinia triticina* Erikss. (*Pt*), is a major foliar disease that poses a significant threat to wheat production in Henan Province, a major wheat-growing region of China. Elucidating the population genetic structure of *Pt* is critical for predicting pathogen dispersal trends and guiding disease management. In this study, 384 *Pt* isolates collected from 13 locations in Henan were genotyped using 11 polymorphic simple sequence repeat (SSR) loci. A total of 204 multilocus genotypes (MLGs) were identified. The Sanmenxia (SMX) population exhibited the highest genetic diversity (*H* = 3.42; *G* = 30.12; *λ* = 0.967), while Shangqiu (SQ) showed the lowest (*H* = 2.09; *G* = 4.59; *λ* = 0.782). Analysis of molecular variance (AMOVA) revealed that 87% of the total genetic variation occurred within populations and 13% among populations. Population structure analyses consistently separated the 13 populations into two genetic clusters, with Xinyang (XY) and SQ forming one distinct group and the remaining 11 populations from the western, central, and northern regions constituting the other. The relative migration network further supported this pattern, showing a highly interconnected network among the central and western populations, but with XY and SQ forming an isolated subnetwork.

## 1. Introduction

Wheat, the most extensively cultivated cereal crop worldwide, serves as a staple food for an estimated 35% of the global population and significantly contributes to global caloric and protein intake [1]. Therefore, it is essential to stabilize wheat production for food and economic security. However, wheat crop is continuously under the threat of diseases and pests [2,3,4]. One of the most significant of these is leaf rust, a fungal disease caused by *Puccinia triticina* Erikss. & Henn. (*Pt*). This pathogen is prevalent in most wheat-growing regions worldwide, with particularly high incidence in Southeast and Central Asia, Eastern Europe, Africa, North America, South America, and Australia [5,6]. The disease severely undermines wheat productivity by damaging photosynthetic leaf tissue, which impairs grain development and filling. This typically results in substantial yield reduction and diminished grain quality, establishing leaf rust as a major biotic constraint on global wheat production [7,8]. Yield losses due to the disease typically range from 7% to 30% [8]. Under environmental conditions favourable for disease development, these losses can exceed 50% [8].

As the foremost global producer and consumer of wheat, China faces significant threats from wheat leaf rust [9,10,11]. Historically, moderate epidemics in northern winter wheat regions during 1969, 1973, 1975, and 1979 resulted in substantial production declines [12]. More recently, severe outbreaks occurred in 2008, 2009, 2012, 2013, and 2015, with the 2012 epidemic being particularly devastating, leading to estimated losses of approximately 3 million metric tons from over 15 million hectares of wheat [13,14,15]. These recurring epidemics highlight the critical need for sustained pathogen surveillance and the development of enhanced management strategies to secure national wheat production against this pervasive fungal disease.

In China, the occurrence of wheat leaf rust exhibits regional specificity, typically affecting the North China Plain, the middle and lower reaches of the Yangtze River, and the southwestern and northeastern regions [11,12]. Henan Province, located in the central part of China, is a key wheat-producing region, contributing roughly one-quarter of the national total, with a planting area of over 5 million hectares [16]. The monoculture of wheat in the province provides an abundant and continuous host environment for the epidemic spread of *Pt*, rendering Henan a prevalent region for this disease [12]. Epidemiological evidence supports the hypothesis that *Pt* lacks suitable conditions for local oversummering in Henan, implying that annual epidemics are likely sustained by long-distance inoculum dispersal [17]. Consequently, managing the disease is challenging due to this external dependency, which also positions Henan as a key component in the larger regional disease dynamic.

Investigating the genetic diversity and population structure of *Pt* is essential for understanding its evolution, identifying inoculum sources, and tracing transmission pathways [18,19]. Over the past few decades, such research has utilized a series of molecular markers, evolving from early methods such as random amplified polymorphic DNA (RAPD) [20] and amplified fragment length polymorphism (AFLP) [21] to the now widely used SSR markers [19,22,23,24,25,26,27] and more recently, SNP-based genomic approaches [28,29]. These studies have revealed high levels of genetic diversity, complex population structure, and measurable gene flow in *Pt*, with spatial patterns varying across geographical scales. Within China, nationwide analyses indicate that *Pt* populations from Henan and Shandong are closely related [30], and significant gene flow has been documented between Henan and several neighboring provinces, including Hebei, Shandong, Inner Mongolia, Jiangsu, Anhui, and Hubei [19,30]. Genetically, Henan clusters within an Eastern population group encompassing eight provinces: Heilongjiang, Beijing, Hebei, Henan, Shanxi, Anhui, Shandong, and Shaanxi [23]. From a north–south differentiation perspective, Henan is categorized within the Northern population group, north of the Qinling Mountains-Huaihe River line, which exhibits lower genetic diversity compared to Southern populations [31].

These large-scale, province-level studies position Henan as a potential hub within the regional dispersal network of *Pt* in eastern China [19,23,31]. However, such a broad scale likely obscures finer-scale genetic structure and localized dispersal dynamics within major wheat-producing provinces. Furthermore, how are the known inter-provincial dispersal pathways of the pathogen specifically realized within Henan Province, and do identifiable intra-provincial dispersal corridors or key nodal regions exist? Elucidating the fine-scale population genetic structure within the province is essential for accurately modeling disease spread and informing regional management strategies.

In the present study, we collected *Pt* isolates from 13 regions across Henan Province, including several regions that had not been previously examined at this level of spatial resolution. This study aimed to elucidate the genetic diversity and population subdivision of *Pt* at a finer spatial scale within Henan, and infer potential intra-provincial dispersal corridors. The results are expected to enhance our understanding of leaf rust epidemiology in this key wheat-producing region and to provide a scientific foundation for targeted disease forecasting and regional deployment of resistant wheat cultivars.

## 2. Materials and Methods

### 2.1. Sample Collection and Reproduction

Samples of *Pt* were collected from wheat leaves exhibiting typical leaf rust symptoms in naturally infected fields across 13 regions in Henan province, China, during the wheat leaf rust outbreak from May to June 2023. Region abbreviations were as follows: Sanmenxia (SMX), Nanyang (NY), Pingdingshan (PDS), Luoyang (LY), Anyang (AY), Hebi (HB), Zhengzhou (ZZ), Shangqiu (SQ), Kaifeng (KF), Xinxiang (XX), Luohe (LH), Xuchang (XC) and Xinyang (XY). At each location, 40 infected leaves were gathered, ensuring representativeness by collecting only 2 to 5 leaves from each field plot. The single-leaf tissue samples were then placed in sulfuric acid paper bags and kept in a desiccated environment at 4 °C for subsequent analysis. Single-uredinial isolates of *Pt* were obtained by selecting a single uredinium, following the method described by Liang et al. [32]. Ten days after planting, seedlings of the susceptible wheat cultivar Mingxian 169 were inoculated with these single-uredinial isolates. Approximately 10–15 days post-inoculation, urediniospores were harvested from the infected wheat leaves for DNA extraction.

### 2.2. DNA Extraction and SSR Amplification of Pt

The genomic DNA of *Pt* from urediniospores was extracted by modified cetyltrimethylammonium bromide (CTAB) method [33]. Additionally, DNA of the leaf rust fungus was extracted from a subset of infected leaf tissues (approximately 2 cm in length) following the protocol described by Ali et al. [34]. The samples of DNA, once extracted, were stored at −20 °C until use.

A set of 11 microsatellite markers was used to genotype the samples (Table S1). Among these primers, RB1, RB8, RB11, RB19 and RB35 were developed by Duan et al. [35], while PtSSR61A, PtSSR68, PtSSR151A, PtSSR161, PtSSR164 and PtSSR173 were previously described by Szabo and Kolmer [36]. The primers were modified with fluorescent dyes FAM, HEX, or TAMRA (General Biol Co., Ltd., Anhui, China) at the 5′ end synthesized by General Biol Co, Ltd. (Anhui, China). PCR amplifications were carried out in a 10 µL volume containing 1.0 µL DNA (5–20 ng/µL), 0.5 µL of each primer (10 mM), 5 µL of 2×UltraTaq PCR StarMix (Kangrun Chengye Biotechnology Co., Ltd., Beijing, China), and 3 µL ddH_2_O. The thermal cycling protocol consisted of an initial denaturation step at 94 °C for 4 min, followed by 35 cycles of denaturation at 94 °C for 30 s, annealing at 56–64 °C (depending on the primer set) for 30 s, and extension at 72 °C for 1 min, with a final extension at 72 °C for 10 min.

Following confirmation via gel electrophoresis, PCR products were sent for capillary electrophoresis analysis on an ABI 3730xl DNA Analyzer (Applied Biosystems, Norwalk, CT, USA) at General Biol Co. Ltd. (Anhui, China). Subsequently, fragment sizing was performed automatically in GeneMapper v4.0 software against a Salmon500 internal standard (65–500 bp). As *Pt* is diploid in the uredinial stage [6], two alleles were scored per isolate to assess homozygosity or heterozygosity at each SSR locus. Finally, a dataset of microsatellite allele sizes was compiled in Microsoft Excel, formatted for subsequent population genetics analysis in GenAlEx v6.5 [37].

### 2.3. Genetic Diversity Analysis

Based on geographical distribution, the samples of *Pt* from Henan province were categorized into 13 populations. Population genetic analyses were conducted using the R package Poppr (version 2.9.8) [38]. Initially, a genotype accumulation curve generated within Poppr was utilized to evaluate the adequacy of the selected SSR loci for distinguishing among all unique individuals. Multiple diversity indices were then calculated, including the number of multilocus genotypes (MLGs) and expected MLGs (*eMLGs*), Stoddard and Taylor’s index *G*, Shannon–Wiener index of MLG diversity *H*, Simpson’s index lambda and evenness *E5*. Additional genetic parameters, including the number of alleles (*Na*), effective number of alleles (*Ne*), Shannon’s information index (*I*), observed heterozygosity (*Ho*), and expected heterozygosity (*He*), were estimated using GenAlEx v6.5 [37], while the average polymorphic information content (*PIC*) was determined with PowerMarker version 2.2.3 [39].

### 2.4. Genetic Differentiation and Population Structure of Puccinia triticina

To investigate genetic differentiation and population structure, we applied multiple approaches to explore. First, an analysis of molecular variance (AMOVA) was carried out using GenAlEx v. 6.5 [37] to estimate genetic variation within and among the 13 *Pt* populations, with significance assessed based on 999 permutations. Additionally, cluster analysis was performed by analyzing the Nei’s genetic distance between samples based on their genetic profile, followed by the construction of an unweighted pair group method with arithmetic mean (UPGMA) dendrogram using the R package Poppr [38].

Local Fisher discriminant analysis (LFDA) was performed using the R package *DA* [40] to infer population genetic structure and visualize the spatial distribution of genetic variation among individuals from different sampling sites.

Discriminant analysis of principal components (DAPC) was implemented using the R package adegenet [41] to infer genetic clusters through a multivariate approach. The optimal number of principal components was selected via cross-validation using the xvalDapc function (1000 replicates; 90% training set) as described by Jombart et al. [42]. The best-supported number of genetic clusters was identified via *K*-means clustering combined with the Bayesian information criterion (BIC), following Jombart et al. [42]. Final visualization was produced using the scatter.dapc function. In addition, the proportions of different molecular groups (MGs) in each sampling location population were summarized and presented as pie charts overlaid on a geographical map to illustrate spatial genetic patterns.

Population genetic structure was further explored using a Bayesian clustering approach in STRUCTURE v2.3.4 [43]. Analyses were run for *K* values ranging from 1 to 10, with 10 replicates per *K*. Each run included a burn-in of 10,000 iterations followed by 100,000 Markov Chain Monte Carlo (MCMC) repetitions. The optimal *K* was determined using the Δ*K* method [44] via the online tool StructureSelector [45], and individual membership coefficients for each *K* were visualized following the CLUMPAK pipeline [46].

Finally, a directional relative migration network among the sampling locations in Henan Province, China, was constructed using the effective number of migrants (*Nm*) as a differentiation index in divMigrate-online [47,48]. This approach generates network plots that effectively visualize gene flow patterns between locations.

## 3. Results

### 3.1. SSR Polymorphisms

**Figure 1:**
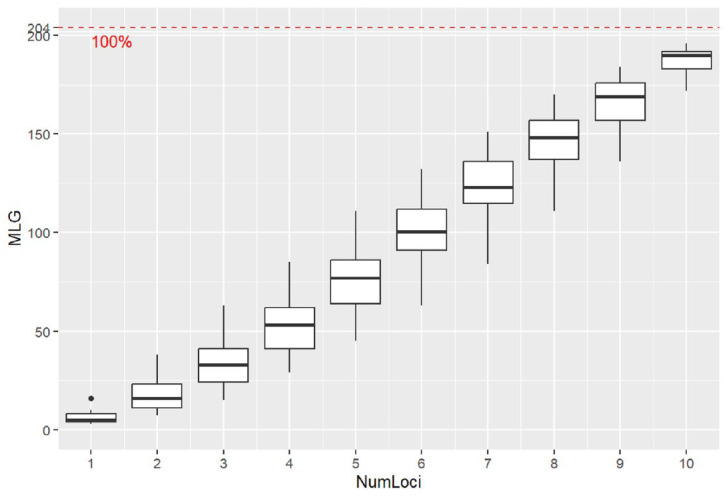
Genotype accumulation curve of different numbers of primers. Note: The black dot denotes an outlier. When the genotype curve showed 100% indicating the number of primer pairs enough to analyze all the MLGs.

The genotype accumulation curve displays a plateau upon reaching ten randomly sampled loci (Figure 1). The curve indicates that the eleven loci employed in our study were sufficient for distinguishing all observed MLGs (Figure 1). A genotype is the unique set of alleles an individual has at specific genetic locations. Individuals with the same alleles at these locations have the same MLG.

All 11 SSR loci utilized in this study exhibited polymorphism, with an average of 4.36 observed alleles (Table 1). While PtSSR151 and RB1 displayed only two alleles, the remaining SSR markers generated between three and nine microsatellite alleles. The diversity indices, *1-D* and *uHe*, showed comparable values, with *1-D* ranging from 0.13 to 0.64 and *uHe* ranging from 0.12 to 0.62. The evenness of allele distribution varied between 0.42 and 0.95. Notably, RB29 exhibited the highest diversity, with a *1-D* value of 0.64, whereas RB11 demonstrated the most uniform allele distribution, achieving an evenness of 0.95. In contrast, the PtSSR61 locus displayed a low level of diversity, with a *1-D* value of 0.13 and uHe of 0.12, alongside an uneven allele distribution characterized by an evenness score of 0.46. The mean polymorphic information content (*PIC*) ranged from 0.12 at locus PtSSR61 to 0.58 at RB29 (Table 1).

### 3.2. Genetic Diversity

A total of 204 MLGs were identified from 384 *Pt* isolates collected from 13 sampling locations in Henan Province, China (Table 2). Based on the *eMLG* values, the SMX population exhibited the highest number of *eMLGs*, while the *eMLG* counts in other populations exceeded 11, suggesting a relatively high level of genotype richness among the populations in Henan Province. Among the 13 populations studied, SMX displayed the highest genotype diversity indices, with a Shannon–Wiener index (*H*) of 3.42, a Stoddart and Taylor index (*G*) of 30.12, and a Simpson index (*λ*) of 0.967, followed closely by NY with indices of *H* = 3.34, *G* = 26.95, and *λ* = 0.963. Conversely, the SQ population exhibited the lowest genotype diversity parameters, with *H* = 2.09, *G* = 4.59, and *λ* = 0.782. Additionally, the high evenness observed in the populations from SMX (*E.5* = 0.982) and NY (*E.5* = 0.957) indicates that genotypes are distributed evenly within these populations, reflecting a greater level of genotype diversity (Table 2). At the population level, the values for the number of alleles (*Na*), effective number of alleles (*Ne*), and Shannon’s diversity index (*I*) were 2.762, 1.947, and 0.706, respectively. Analysis of intra-population genetic variance across the 13 regions revealed that Na ranged from 2.545 to 3.091, *Ne* from 1.772 to 2.097, and *I* from 0.598 to 0.795, indicating a relatively high level of genetic diversity among populations. Notably, the SQ population exhibited the lowest genetic diversity, with an I value of 0.598. Observed heterozygosity (*Ho*) was higher than expected heterozygosity (*He*) in all 13 populations. *Ho* ranged from 0.548 (SQ) to 0.733 (LH) with a global value of 0.643. *He* ranged from 0.363 (SQ) to 0.489 (XX) with a global value of 0.426.

### 3.3. Genetic Differentiation and Population Structure

According to the AMOVA, 87% of the total genetic variation among the 384 isolates was attributed to differences within populations, while only 13% originated mainly from variation among populations (Table 3).

**Figure 2:**
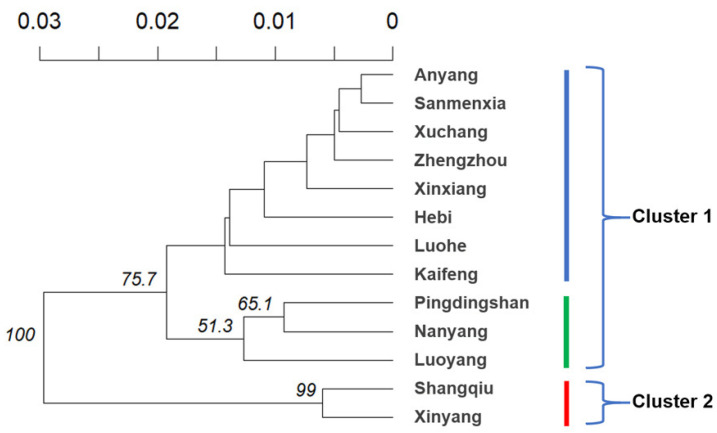
The UPGMA Trees generated on regional levels based on Nei’s genetic distance. Note: Unweighted pair group method with arithmetic mean (UPGMA) clustering of *Puccinia triticina* population from 13 sampling locations in Henan province of China based on Nei’s genetic distance. Bootstrap values >50% (1000 replicates) are shown at nodes.

On the basis of Nei’s genetic distance, UPGMA-based cluster analysis was performed to understand the relationships between the 13 populations from sampling locations in Henan province of China. As a result, all 13 populations were grouped into two major clusters when the distance was 0.02, as shown in Figure 2. The cluster 1 was further subdivided into two subclusters. AY and SMX populations converged first, then gathered with XC, ZZ, XX, HB, LH, and KF populations. In the other subcluster, PDS, NY, and LY were clustered as a group. The remaining SQ and XY populations formed cluster 2.

**Figure 3:**
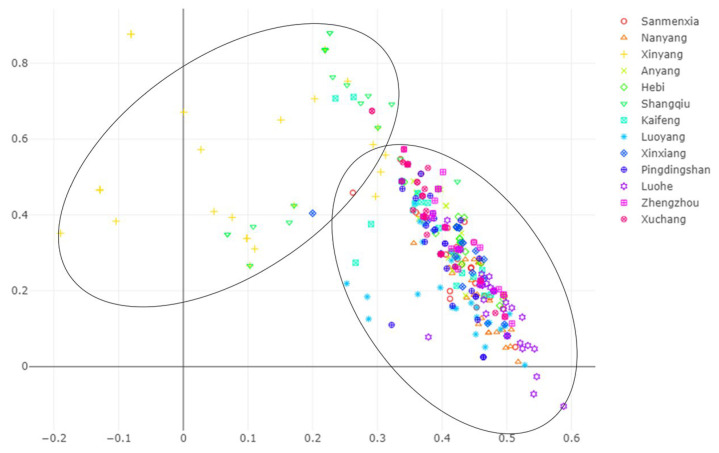
Two-dimensional LFDA projection revealing the population genetic structure of 13 *Puccinia triticina* populations from Henan Province, China.

LFDA revealed clear genetic structure across the sampled populations (Figure 3). The left cluster was dominated by individuals from XY and SQ, with little admixture from other locations, indicating strong genetic distinctiveness of these eastern populations. In contrast, the right cluster encompassed all remaining sampling sites, with individuals showing a continuous genetic cline that broadly correlated with their geographic proximity. Notably, populations in the central and western regions (e.g., ZZ, XC, LH, and PDS) exhibited substantial genetic overlap, reflecting high gene flow and weak genetic differentiation among these geographically proximate sites.

**Figure 4:**
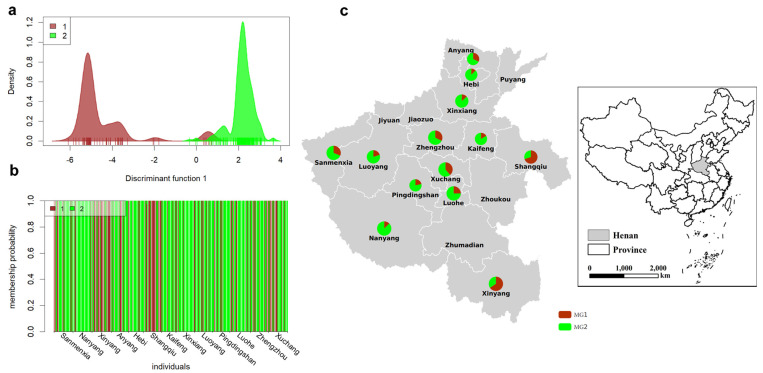
Discriminant analysis of principal components (DAPC) reveals two genetic groups (MG1 and MG2) of target organism populations in Henan Province, China. (**a**) Density scatter plot of DAPC. (**b**) Individual membership probability bar plot. (**c**) The main map displays Henan Province, with pie charts at each sampling site showing the proportion of individuals assigned to MG1 (brown) and MG2 (green).

Nonparametric DAPC analysis was conducted to further investigate the genetic clusters. Based on cross-validation analysis, we retained 24 principal components from PCA during the preliminary data transformation step to infer the number of clusters. The optimal number of clusters was determined to be two. When *K* = 2, such as shown in Figure 4a indicate that the separation effect of the MGs is optimal, with virtually no overlap between the brown and green MGs. The distributions of these MGs in the geographic regions are shown in Figure 4c. the brown MG1 had a significant proportion in XY and SQ. The green MG2 was significant in NY, HB and XX, and it was also presented in other populations (Figure 4c). The presence of the same MGs in different geographic regions of Henan Province suggested the pathogen may have migrated between regions.

**Figure 5:**
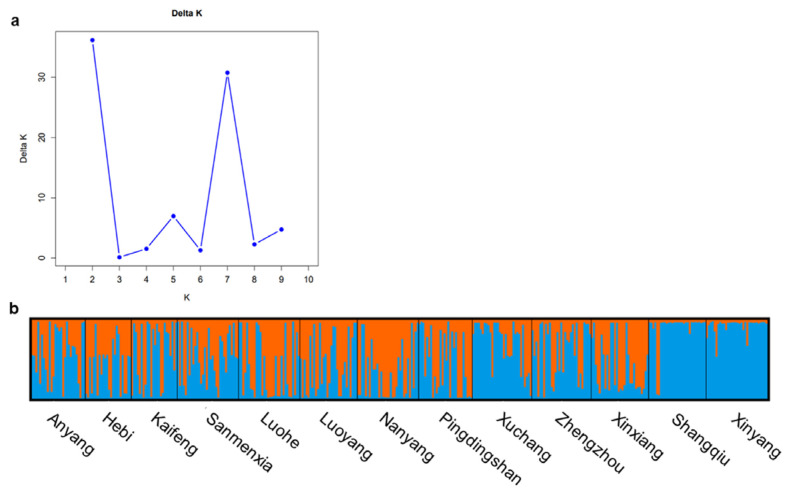
The population genetic structure of 384 *Puccinia triticina* isolates from 13 locations in Henan, China, was inferred using the Bayesian algorithm implemented in STRUCTURE v2.3.4. (**a**) The plot shows the selection of the optimal cluster number (*K*) based on Δ*K* values. (**b**) Each vertical bar represents a single sampled individual. The colors (orange and blue) correspond to the two genetic clusters identified by *K* = 2.

Based on Bayesian modeling in STRUCTURE, the optimal number of clusters was determined to be *K* = 2 (Figure 5). The resulting bar plot at *K* = 2 revealed two genetic clusters. One was uniquely formed by the XY and SQ populations and exhibited limited admixture, whereas the other included all 11 remaining populations and showed widespread evidence of mixed ancestry.

### 3.4. Directional Genetic Differentiation and Relative Migration Between Puccinia triticina Populations

**Figure 6:**
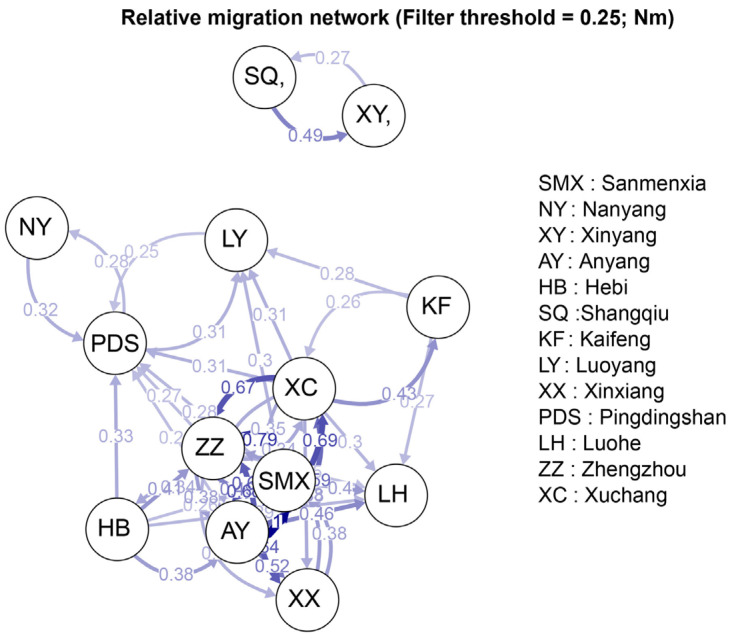
Directional relative migration network among *Puccinia triticina* populations in Henan Province, China. The network was inferred using divMigrate-online based on Nm statistics (filter threshold = 0.25). Circles denote the 13 sampling locations, and arrows indicate the direction and magnitude of relative migration, with darker colours representing higher migration rates and illustrating stronger gene flow between geographically proximate populations.

The directional relative migration network of *Pt* populations showed high connectivity among sampling locations, particularly within SMX, AY, ZZ, XC, and XX, which were interconnected through multiple migration pathways (Figure 6). These nodes demonstrated bidirectional genetic exchanges, both receiving and contributing migrants to adjacent populations. The highest level of relative migration was observed between SMX and AY, with values of 1.0 and 0.9, respectively. Notably, XY maintained significant gene flow exclusively with SQ, forming an isolated subnetwork that remained distinct from other geographical groups (Figure 6).

## 4. Discussion

Understanding the population genetics of phytopathogens enhances our insight into their biology, evolution, migration, gene flow, and adaptation, which can be applied to develop and refine disease management strategies [49]. In the present study, *Pt* populations in Henan Province exhibited a distinct genetic pattern characterized by high within-population variation and low between-population differentiation. AMOVA results indicated that the majority of genetic variation (87%) originated within populations, whereas differentiation among populations was relatively weak (13%) (Table 3). This finding aligns with previous reports on *Pt* populations from multiple provinces in China [19,29,31]. In the LFDA plot, populations from central and western regions exhibited substantial genetic overlap, with the exception of XY and SQ populations, indicating strong gene flow and weak genetic differentiation among these geographical populations (Figure 3). Sustained high levels of gene flow can homogenize genetic differences between populations, counteracting the divergence caused by genetic drift or local adaptation. Additionally, the divMigrate-based network in this study reveals relative genetic connectivity among geographic populations, which is consistent with frequent genetic exchange between populations (Figure 6). This broad pattern of gene flow suggests relatively unimpeded dispersal pathways for *Pt* populations across Henan Province, and may provide a genetic basis for the cross-regional colonization and adaptive persistence of this phytopathogen within the region.

Although the overall differentiation level is low, multiple population genetic analyses consistently identified the optimal number of clusters as two (*K* = 2). UPGMA clustering divided the 13 populations into two major groups at a genetic distance of 0.02. In this analysis, the SQ and XY populations independently formed Cluster 2, whereas the remaining 11 populations grouped into Cluster 1, which was further subdivided into two distinct subclusters (Figure 2). Similarly, DAPC and STRUCTURE analyses under the optimal *K* = 2 also clearly revealed a substructure, with the XY and SQ populations forming a separate cluster and the other 11 populations from western, central, and northern areas grouping into another major cluster (Figure 4 and Figure 5). These consistent results indicate the presence of a well-defined genetic structure among *Pt* populations in Henan Province. Based on previous published studies and geographic plausibility, we infer that the formation of this genetic structure may be associated with inoculum sources [17,19,23,29,31,50].

Henan is part of the spring epidemic region for wheat leaf rust, where local inoculum rarely survives the extreme summer heat [17]. Thus, initial seasonal infections rely largely on external inoculum sources [19,23,31]. Li et al. [31] confirmed a south-to-north dissemination pathway for wheat leaf rust in eastern China. In this pathway, southern wheat-producing regions such as Jiangsu and Anhui provinces, characterized by warm and humid climates, serve as inoculum sources of *Pt*. Urediniospores of *Pt* are dispersed northward by southerly winds to Henan and Shandong provinces, and eventually infect wheat plants in Hebei Province and Beijing Municipality. XY (southern Henan) and SQ (eastern Henan) border Hubei Province and Anhui Province, respectively, thus becoming the first regions within Henan to receive inoculum input from southern sources. These geographic and epidemiological characteristics result in significant differences in genetic composition of *Pt* populations between XY and SQ and other regions of Henan Province.

Directional relative migration network analysis recovered patterns of asymmetric genetic connectivity among the 11 *Pt* populations from western, central, and northern Henan (Figure 6). Populations from SMX, PDS, ZZ, and XC showed the highest number of pairwise connections and elevated relative migration coefficients (Figure 6), and may function as putative hubs of genetic exchange. The SMX population exhibited higher relative migration coefficients toward central Henan populations (Figure 6). In turn, central Henan populations, particularly ZZ and XC, displayed strong connectivity with multiple surrounding populations, consistent with a role as relay points for genetic exchange. These patterns are consistent with a putative west-to-east gradient of gene dispersal for *Pt* within Henan Province. These genetic connectivity patterns are congruent with findings from previous regional studies that reported weak genetic differentiation between Henan populations and those from adjacent Shaanxi and Gansu provinces [29]. Prior work by Li et al. [50], based on aerobiological and epidemiological evidence, has suggested that *Pt* inoculum from Gansu and Shaanxi may disperse eastward into western Henan via wind currents and geographic corridors during the spring epidemic period. Taken together, our population genetic data and published evidence support the plausibility of Henan serving as a key intermediate zone in the eastward spread of *Pt* inoculum from western source areas, though direct empirical confirmation would require temporal sampling and aerobiological monitoring.

LFDA weights genetic relatedness based on local neighborhood relationships, thereby maximizing inter-population differentiation while effectively preserving multi-modal genetic structure, and remains robust even in the presence of subtle genetic clines and admixture [40,51]. LFDA results reveal a divergent trend between the eastern populations (XY and SQ) and the other populations, which substantiates the contribution of *Pt* inoculum sources to shaping the spatial genetic structure of the pathogen (Figure 3). This observation aligns with previous studies that have validated the capacity of LFDA to identify such genetic distribution patterns using both simulated and empirical datasets [52].

SSR analysis revealed considerable genetic diversity within Henan *Pt* populations. A total of 204 MLGs were identified from 384 *Pt* isolates collected across 13 sampling sites. After rarefaction correction, the expected MLG (*eMLG*) count for each region exceeded 11 (Table 2), indicating a high reservoir of genotypes even after sample size standardization, which is substantially higher than reports from some single ecological regions [19,23,31]. At the population level, the observed number of alleles (*Na* = 2.762), effective number of alleles (*Ne* = 1.947), and Shannon’s information index (*I* = 0.706) were all at medium-to-high levels compared to *Pt* populations in other major wheat-producing regions of China [19,31]. As aforementioned, Henan, as a confluence hub, continuously receives diverse inoculum from eastern, western, and southern sources [19,23,31,50]. These strains with different genetic backgrounds provide a fundamental and rich reservoir of genetic variation for local populations. Beyond external introduction, intrinsic pathogen evolution mechanisms significantly contribute to regional diversity. Recent genomic evidence has established somatic nuclear exchange as a key driver of genetic diversification in clonally propagating *Pt* populations [53]. This process facilitates the exchange of haploid nuclei between genetically distinct strains within a shared host, rapidly generating novel dikaryotic combinations and accelerating local adaptive variation. In Henan, where multiple pathogen lineages converge, the frequency of such nuclear exchange events is likely elevated, further amplifying regional genetic diversity.

The population genetic structure and diversity characteristics of *Pt* in Henan Province revealed in this study provide essential genetic background to inform the formulation of regional wheat leaf rust management strategies. For regions with genetically distinct pathogen populations (e.g., the XY and SQ populations that act as primary entry points for southern inoculum), enhanced monitoring of incoming inoculum sources and their virulence profiles should be prioritized to address the potential risks posed by diverse introduced pathogen lineages. For the 11 populations across western, central, and northern Henan that show high genetic connectivity and frequent gene flow, strengthened cross-regional collaborative monitoring is critical to track the dynamics of pathogen dispersal and genetic exchange between Henan and neighboring provinces.

## 5. Conclusions

This study revealed high genetic diversity within the *Pt* population in Henan Province, with 87% of the genetic variation residing within populations. UPGMA clustering, DAPC, and STRUCTURE analyses consistently grouped the 13 geographic populations into two major clusters: XY and SQ formed an isolated, genetically distinct cluster, whereas the remaining 11 populations from central and western Henan displayed extensive admixture. Directional migration analysis confirmed strong gene flow and bidirectional migration among most locations, while XY and SQ remained an isolated subnetwork. These findings highlight the necessity of region-specific disease surveillance and targeted resistance breeding to achieve effective management of wheat leaf rust in this major wheat-producing area.

## Figures and Tables

**Table 1:** Number of Allele, *1-D*, *uHe* and Evenness of the 11 microsatellite loci isolated from *Puccinia triticina*.

Locus	Allele	*1-D*	*uHe*	Evenness	*PIC*
PtSSR68	9	0.63	0.62	0.74	0.56
PtSSR161	5	0.22	0.21	0.42	0.22
PtSSR173	5	0.62	0.60	0.87	0.55
PtSSR61	4	0.13	0.12	0.46	0.12
PtSSR151	2	0.40	0.38	0.83	0.32
PtSSR164	3	0.63	0.62	0.93	0.55
RB1	2	0.45	0.44	0.91	0.35
RB8	3	0.46	0.46	0.90	0.36
RB11	3	0.51	0.51	0.95	0.39
RB29	8	0.64	0.61	0.71	0.58
RB35	4	0.32	0.30	0.64	0.27
mean	4.36	0.46	0.44	0.76	0.39

Note: *1-D*, Simpson index; *uHe*, Nei’s 1978 gene diversity; *PIC*, polymorphic information content.

**Table 2:** Genetic diversity information of different *Puccinia triticina* populations.

Population	N	MLG	*eMLG*	*G*	*H*	*λ*	*E.5*	*Na*	*Ne*	*Ho*	*He*	*I*
Sanmenxia (SMX)	32	31	23.4 ± 0.497	30.12	3.42	0.967	0.982	2.909	1.938	0.648	0.438	0.712
Nanyang (NY)	32	29	22.3 ± 0.810	26.95	3.34	0.963	0.957	3.091	1.920	0.580	0.453	0.742
Xinyang (XY)	32	16	13.4 ± 1.144	9.48	2.50	0.895	0.755	2.545	1.883	0.574	0.406	0.665
Anyang(AY)	28	21	18.8 ± 0.894	17.82	2.97	0.944	0.912	2.636	1.882	0.617	0.410	0.663
Hebi (HB)	24	16	16.0 ± 0.000	10.29	2.57	0.903	0.771	2.545	1.841	0.652	0.406	0.645
Shangqiu (SQ)	30	14	11.9 ± 1.042	4.59	2.09	0.782	0.508	2.545	1.772	0.548	0.363	0.598
Kaifeng (KF)	24	20	20.0 ± 0.000	16.94	2.93	0.941	0.904	2.545	1.948	0.663	0.437	0.691
Xinxiang (XX)	30	28	22.6 ± 0.654	25.00	3.29	0.960	0.927	3.091	2.045	0.655	0.489	0.795
Luoyang (LY)	30	18	15.5 ± 1.069	12.50	2.71	0.920	0.821	2.818	2.007	0.685	0.449	0.734
Pingdingshan (PDS)	28	22	19.4 ± 0.865	17.04	2.99	0.941	0.853	2.818	2.004	0.662	0.471	0.761
Luohe (LH)	32	25	19.8 ± 1.091	19.69	3.12	0.949	0.864	2.909	2.097	0.733	0.463	0.754
Zhengzhou (ZK)	31	23	19.1 ± 1.073	19.61	3.06	0.949	0.916	2.727	2.028	0.692	0.449	0.729
Xuchang (XC)	31	18	14.9 ± 1.140	9.91	2.61	0.899	0.710	2.727	1.952	0.648	0.430	0.691
Total	384	204	21.3 ± 1.550	67.27	4.84	0.985	0.530	2.762	1.947	0.643	0.426	0.706

Abbreviations: N, number of individuals observed; *Na*, observed number of alleles; *Ne*, effective number of alleles; MLG, number of multilocus genotypes (MLGs) observed; *eMLG*, the number of expected MLG at the smallest sample size ≥10 based on rarefaction; *H*, Shannon–Wiener Index of MLG diversity; *G*, Stoddart and Taylor’s Index of MLG diversity. *λ*, Simpson’s Index; *E.5*, evenness; *I*, Shannon’s Information index; *Ho*, observed heterozygosity; *He*, expected heterozygosity.

**Table 3:** The analysis of molecular variance (AMOVA) for *Puccinia triticina* populations from 13 sampling locations in Henan province of China.

Source	df	SS	%	*Φpt*	*p*
Among Pops	12	164.634	13	0.126	0.001
Within Pops	371	969.822	87		
Total	383	1134.456	100		

## Data Availability

The raw data are available from the corresponding author upon reasonable request.

## Supplementary Materials

The following supporting information can be downloaded at https://www.mdpi.com/article/10.3390/jof12070468/s1, Table S1: List of simple sequence repeat (SSR) used in the study.
