# Supplementary material for: Genetic Analysis Reveals Relationships Among Populations of Puccinia triticina from Henan Province of China
Source: J Fungi (Basel). 2026 Jun 25;12(7):468. doi: 10.3390/jof12070468 (PMC13412825; doi:10.3390/jof12070468)
Supplement: Supplementary file 1 [file jof-12-00468-s001.zip › jof-4348496-supplementary.pdf]

Table S1 List of simple sequence repeat (SSR) used in the study

ID	Locus	Repeat motif	Primer sequence (5' to 3')	5' Florescence	Alleles	Annealing temperature (℃)	Allele size Range (bp)
1	RB1F<br>RB1R	$(GT)_5$	TTGTCGTTCTGGAATGATGC<br>TGCCCACAACCCTCCTC	TAMRA	2	56	126-134
2	RB8F<br>RB8R	$(TGG)_7$	CGCCGTTCCCATCGTTC<br>TAAAACACTCCACCCACGCC	HEX	3	64	138-141-147
3	RB11F<br>RB11R	$(CA)_{17}$	AGCAGTGAGCAGCAGCGTC<br>ACTACTGTGAGTGTCGGCTTGG	FAM	3	58	178-204-208
4	RB29F<br>RB29R	$(CA)_{15}$	CTCACCAAACATCAAGCACC<br>GAGCCTAGCATCAGCATCC	HEX	8	60	106-144
5	RB35F<br>RB35R	$(AC)_9 + (TA)_5 + (AG)_5$	ACTGCGATATCCAGTACACACAC<br>TGATGGGCTCGCAGTGG	FAM	4	62	238-244-246-248
6	PtSSR61F<br>PtSSR61R	$(TG)_9$	CGAACTGGTACAACGCACTG<br>CGCAAAAAGGCTGATCTCTG	TAMRA	4	62	297-303
7	PtSSR68F<br>PtSSR68R	$(TG)_{16}$	GACTCAGCCCACTGCTAACC<br>GATGGCGACGTATTTGGTCT	HEX	9	62	309-333
8	PtSSR151AF<br>PtSSR151AR	$(AAC)_{12}$	TCATCGCACTCCACTCAGAC<br>ATGCTGCCCAACCTGCTC	FAM	2	61	472-475
9	PtSSR161F<br>PtSSR161R	$(TC)_{13}$	ACTGCCTCCTGTGCCTTCT<br>TAGTCCGAGGGTGACGAAGT	TAMRA	4	60	206-216-218-220
10	PtSSR164F<br>PtSSR164R	$(TC)_{13}$	GTGGAAGTGAGCGGAAGAAG<br>GGAGATGGGCAGATGAGGTA	HEX	3	61	216-222-224
11	PtSSR173F<br>PtSSR173R	$(TC)_3 + (TC)_{12}$	CTCAGCGACCTCAAAGAACC<br>GAGACGACGGATGTCAACAA	FAM	5	60	212-220
